# Treatment of Cutaneous Melanoma Harboring SMO p.Gln216Arg Mutation with Imiquimod: An Old Drug with New Results

**DOI:** 10.3390/jpm11030206

**Published:** 2021-03-14

**Authors:** Teresa Troiani, Stefania Napolitano, Gabriella Brancaccio, Valentina Belli, Annarita Nappi, Caterina Miro, Domenico Salvatore, Monica Dentice, Michele Caraglia, Renato Franco, Emilio Francesco Giunta, Vincenzo De Falco, Davide Ciardiello, Fortunato Ciardiello, Giuseppe Argenziano

**Affiliations:** 1 Medical Oncology, Department of Precision Medicine, Università degli Studi della Campania “Luigi Vanvitelli”, 80131 Naples, Italy; stefania.napolitano@unicampania.it (S.N.); valentina.belli@hotmail.com (V.B.); emiliofrancescogiunta@gmail.com (E.F.G.); vincenzodefalc@gmail.com (V.D.F.); davideciardiello@yahoo.it (D.C.); fortunato.ciardiello@unicampania.it (F.C.); 2 Dermatology Unit, Department of Mental and Physical Health and Prevention Medicine, Università degli Studi della Campania “Luigi Vanvitelli”, 80131 Naples, Italy; gabri.brancaccio@gmail.com (G.B.); giuseppe.argenziano@unicampania.it (G.A.); 3 Department of Clinical Medicine and Surgery, University of Naples “Federico II”, 80131 Naples, Italy; annarita.nappi@unina.it (A.N.); caterina.miro@unina.it (C.M.); monica.dentice@unina.it (M.D.); 4 Department of Public Health, University of Naples “Federico II”, 80131 Naples, Italy; domsalva@unina.it; 5 Biochemistry Unit, Department of Precision Medicine, Università degli Studi della Campania “Luigi Vanvitelli”, 81100 Naples, Italy; michele.caraglia@unicampania.it; 6 Pathology Unit, Department of Mental and Physical Health and Prevention Medicine, Università degli Studi della Campania “Luigi Vanvitelli”, 81100 Naples, Italy; renato.franco@unicampania.it

**Keywords:** melanoma, imiquimod, SMO, Hedgehog pathway

## Abstract

Melanoma is the most lethal form of skin cancer and its incidence is growing worldwide. In the last ten years, the therapeutic scenario of this disease has been revolutionized by the introduction of targeted therapies and immune-checkpoint inhibitors. However, in patients with many lesions and bulky tumors, in which surgery is no longer feasible, there is a need for new treatment options. Here we report, for the first time to our knowledge, a clinical case where a melanoma patient harboring the SMO p.Gln216Arg mutation has been treated with imiquimod, showing a complete and durable response. To better explain this outstanding response to the treatment, we transfected a melanoma cell line (MeWo) with the SMO p.Gln216Arg mutation in order to evaluate its role in response to the imiquimod treatment. Moreover, to better demonstrate that the antitumor activity of imiquimod was due to its role in suppressing the oncogenic SMO signaling pathway, independently of its immune modulating function, an in vivo experiment has been performed. This clinical case opens up a new scenario for the treatment of melanoma patients identifying a new potentially druggable target.

## 1. Introduction

Melanoma is the most lethal form of skin cancer and its incidence is growing worldwide, with over 287,000 new cases and over 60,000 deaths in 2018 [1]. The majority of melanoma patients have an early-stage disease at diagnosis, while approximately 10% of cases are diagnosed at an advanced stage and are unresectable or already metastatic [2]. About 20% of relapsed melanoma patients develop satellite and/or in-transit metastases without nodal involvement [3]. Based on the clinical presentation, which can range from a single or a few, to several lesions and/or bulky tumors, different treatments have been tested. In patients with few lesions, complete surgical resection is the curative approach. However, in patients with many lesions and bulky tumors, and in cases where surgery is no longer feasible, there is a need for new treatment options. Several case reports have reported promising results in the treatment of cutaneous melanoma metastases with the use of imiquimod (IMQ) 5% cream as monotherapy [4,5], or in association with cryosurgery [6], topical retinoid plus intralesional interleukin-2 [7], and 5-fluoruracil [8].

IMQ is a synthetic imidazoquinoline amine, whose main mechanism of action includes the modification of immune response through an agonism of toll-like receptors 7 and 8 [9]. This interaction promotes the activation of a signaling cascade, leading to the translocation of nuclear factor-kappa B (NF-κΒ). NF-κΒ binds to DNA and induces the expression of many pro-inflammatory cytokines from the peripheral blood mononuclear cells, such as interferon-α (IFN-α), tumor necrosis factor α (TNF-α), interleukin IL-1, IL-2, IL-6, IL-8, IL-12, granulocyte colony stimulating factor (GM-CSF), granulocyte macrophage colony stimulating factor (GM-CSF), as well as chemokines, such as CCL4 and CCL2. The resulting inflammatory reaction and antitumor response involves plasmacytoid dendritic and cytotoxic CD8+ cells attacking the tumor. Moreover, preclinical studies revealed that IMQ acts as a potent immune response modifier through its ability to induce the production of IFNa and other cytokines, which, in turn, stimulate T cells, thereby enhancing both the innate and adaptive immune system, particularly the cell-mediated pathways [9].

Furthermore, it has been demonstrated that IMQ can also directly repress Hedgehog (HH) and Smoothened (SMO) pathways by negatively modulating GLI activity through adenosine receptors (ADORAs) [10]. IMQ has been approved for the treatment of actinic keratosis and superficial basal cell carcinoma (BCC) [5]. It also has an off-label use in nodular BCC, extramammary Paget disease, Bowen’s disease, and erythroplasia of Queyrat (in situ squamous cell carcinomas) [5,11,12,13].

Here, we first report an interesting case of a melanoma patient harboring the SMO mutation (p.Gln216Arg) c.647A>G and responsive to imiquimod. SMO is a component of the Hedgehog signaling pathway [14]. SMO and Patched (PTCH)1 are transmembrane receptor proteins that belong to the Hedgehog pathway. In the absence of the HH ligand, PTCH1 represses SMO, while upon the binding of HH to PTCH1, this repression is released. This leads to translocation of the GLI transcription factors into the nucleus. This transduction pathway is involved in the embryonal development of hair follicles and sebaceous glands, but it is mostly inactive or poorly active in the adult organism. The Hedgehog pathway maintains the stem cell population and can be activated in normal skin for wound healing. Mutations in PTCH1 or SMO that lead to the constitutive activation of SMO are known to play a role in the carcinogenesis of basal cell carcinoma, glioblastoma, medulloblastoma, and rhabdomyosarcoma [14,15,16]. The Hedgehog signaling pathway is a critical part of embryonic development [16]. In the skin, Hedgehog proteins are involved in maintenance of stem cells, development of hair follicles, development of glands, and regulation of skin growth [17].

## 2. Case Presentation

**Figure 1:**
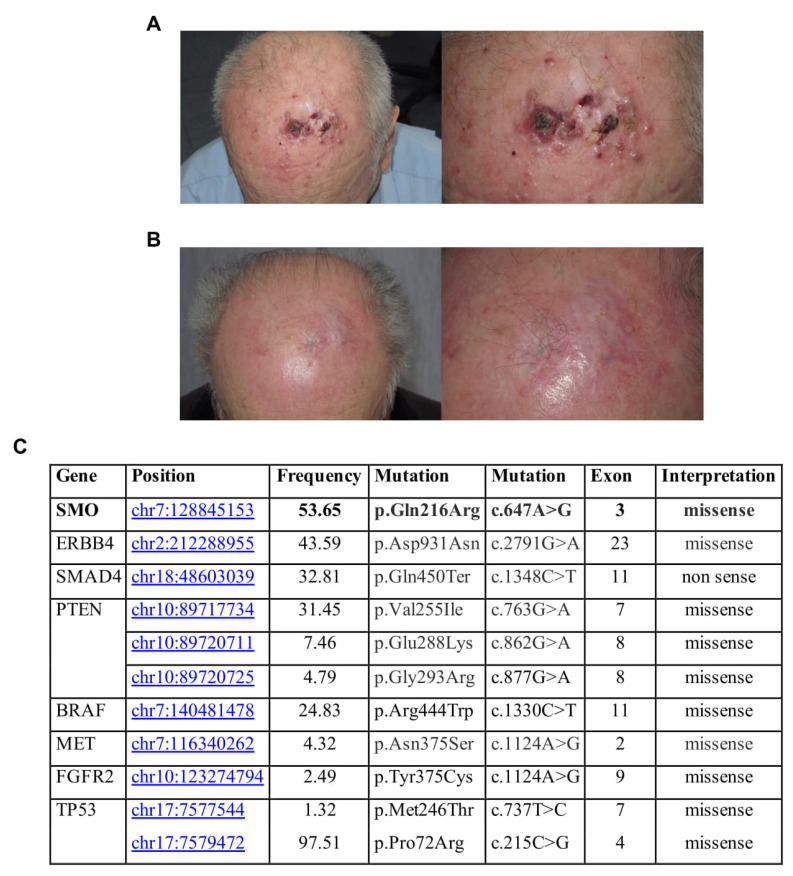
(**A**) Clinical presentation at time of diagnosis; (**B**) Clinical presentation after two months of imiquimod (IMQ) treatment; (**C**) Gene, mutation, frequency, and exon in this sample.

An 85-year-old farmer presented with multiple (up to 25) nodular lesions on the forehead (Figure 1A). History revealed the presence of a non-biopsied tumor in the same location treated with cryosurgery some years before. Histopathologic examination of one of the lesions confirmed the diagnosis of BRAF wild-type melanoma metastases, while staging procedures (CT scan and regional lymph node ultrasound) were negative. The patient, who lives in the countryside far from any referral hospital, refused anti PD-1 therapy, as well as electrochemotherapy. Therefore, we prescribed topical imiquimod 5% cream to be applied locally once daily. Side effects, such as erythema, swelling and pain, led to a brief discontinuation at week 3, but overall, the treatment was well tolerated. After 2 months of treatment, complete regression of the palpable lesions was reached (Figure 1B). Complete clinical resolution was observed after 12 months of follow-up with no disease progression detected through lymph node ultrasound and CT scans. After 32 months clinical resolution was still present, but the patient refused to schedule subsequent clinical surveillance.

To better explain a complete response to the treatment obtained, we analyzed the mutational status of one sample collected by NGS technologies, through an analysis of 2800 mutational hotspots and targeted regions in 50 genes commonly implicated in cancer. NGS analysis showed that this patient had homozygous SMO gene mutation, identified with Novel (p.Gln216Arg) c.647A>G in exon 3 position chr7:128845153. This missense mutation, c.647A>G, with G/A alleles, has unknown functional implications. It is in the Frizzled domain of SMO. Frizzled-containing proteins belong to a family of G protein-coupled receptor proteins that serve as receptors in the Wnt signaling pathway and other signaling pathways. Frizzled domain is responsible for the activation of the downstream signaling. Specifically, this mutation was also found in the basal cell carcinoma, along with other SNPs in the same region associated with an increased risk of BCC development [14]. Mutations affecting SMO could induce an overactivation of the protein and, consequently, a significant clinical response to SMO inhibitors. Additional gene mutations have been found (Figure 1C).

**Figure 2:**
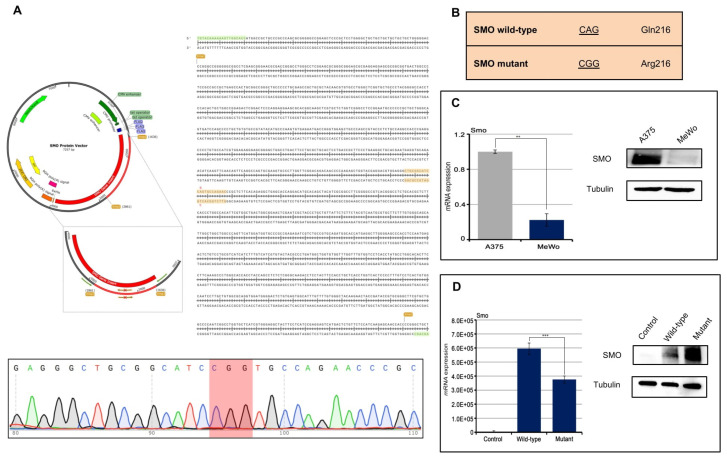
(**A**,**B**) MeWo cell lines were transfected with protein vector for SMO wild-type and SMO mutant (pPM-N-D-C-His) in order to introduce the mutation of interest (CAG vs. CGG); (**C**) Basal SMO expression in A375 cell lines, used as positive control, and MeWo cell lines evaluated by Western Blot assay and qRT-PCR analysis (*p* < 0.01); (**D**) SMO expression was evaluated by Western Blot assay and qRT-PCR analysis (*p* < 0.001) in MeWo cells parental and in the transfected ones. Control: MeWo parental cell line; Wild-type: MeWo cell line transfected with SMO wild-type vector; MeWo cell lines: transfected with SMO mutant vector. ** *p* < 0.01, *** *p* < 0.001.

**Figure 3:**
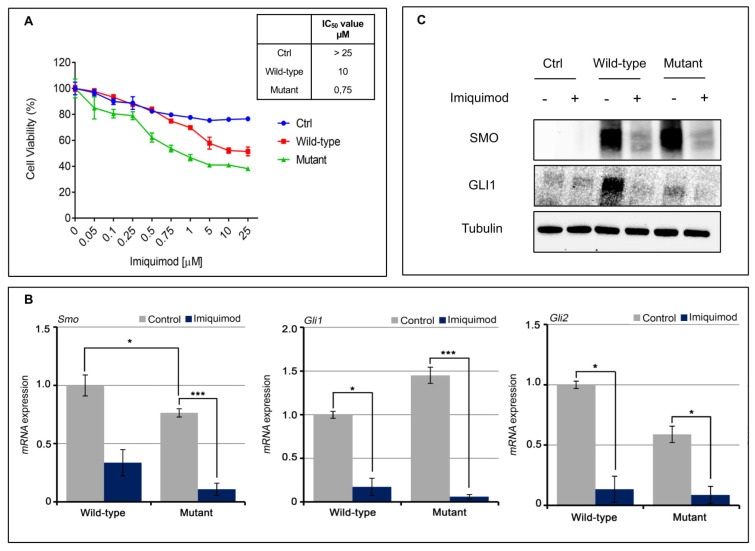
(**A**) MeWO cells were treated with increasing concentration of IMQ for 72 h and cell viability was evaluated by MTT assay. Table represents the IC50 value; (**B**,**C**) Sonic Hedgehog pathways, including SMO, GLI1 and GLI2 were evaluated by western blot (bar graphs in Supplementary Figure S1) and qRT-PCR analysis after IMQ treatment (1 µM). Control: MeWo parental cell line; Wild-type: MeWo cell line transfected with SMO wild-type vector; MeWo cell lines: transfected with SMO mutant vector. * *p* < 0.1, *** *p* < 0.001.

A direct effect of IMQ on the oncogenic HH/SMO pathway in a basal cell carcinoma (BCC) has been reported recently [10]. Here, we transfected a melanoma cell line (MeWo) with the SMO p.Gln216Arg mutation in order to evaluate its role in response to IMQ treatment (Figure 2A,B). Transfection of the SMO gene (wild-type and mutant) results in the cells stably overexpressing the SMO protein (Figure 2C,D). We have exposed the SMO wild-type and mutant gene amplified MeWo cell lines to an increased concentration of IMQ (Figure 3A). As shown in Figure 3a, IMQ showed a strong antiproliferative effect only in mutant-gene-amplified MeWo cells with an IC50 of 0.75 μM. Moreover, IMQ treatment induced a statistically significant reduction in mRNA SMO expression and its effectors GLI1 and GLI2 with a consequent reduction in their protein expression (Figure 3B,C). These results suggest that IMQ may directly repress oncogenic the SMO signaling pathway, independent of its immune-modulating function.

**Figure 4:**
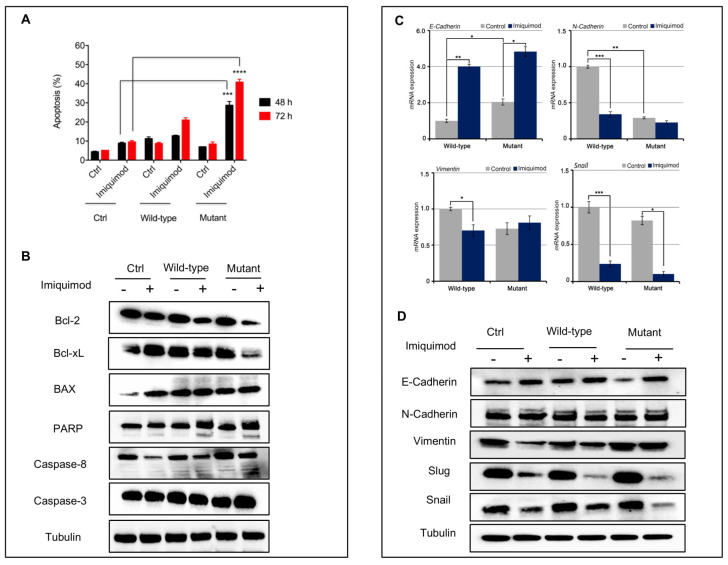
(**A**) Bar chart showing the percentage of apoptotic cells on MeWo cell lines following treatment with imiquimod (1 µM) for 48 and 72 h; (**B**) Apoptotic markers after treatment with IMQ (1 µM) in MeWo cells were evaluated by Western blot analysis (bar graphs in Supplementary Figure S2); (**C**,**D**) EMT markers before and after treatment with IMQ (1 µM) in MeWo cells were evaluated by qRT-PCR and Western blot analysis (bar graphs in Supplementary Figure S3). Control: MeWo parental cell line; Wild-type: MeWo cell line transfected with SMO wild-type vector; MeWo cell lines: transfected with SMO mutant vector. * *p* < 0.1, ** *p* < 0.01, *** *p* < 0.001.

Next, we have measured the ability of IMQ to induce apoptosis by using Annexin V-FITC assay in these models. As depicted in Figure 4A, IMQ determined a significant induction of apoptosis especially in the mutant model. We also tested the expression of the anti-apoptotic components by Western blot analysis. As shown in Figure 4B, we observed a strong reduction in expression levels of BcL2 and in the cleavage of PARP in the mutant-gene-amplified MeWo cells in the presence of IMQ treatment, suggesting a probable activation of anti-apoptotic mechanisms.

In addition, reactivation of the HH pathway with epithelial mesenchymal transition (EMT) activation has been implicated in the carcinogenesis of several cancer types [18], and its inhibition can reverse EMT, enhancing tumor sensitivity to cytotoxic agents [19]. In this work, we assisted with the loss of mesenchymal markers. In particular, we showed a down-regulation of SLUG, SNAIL and Vimentin, and up-regulation of E-cadherin in our models treated with IMQ compared to untreated ones (Figure 4C,D). This effect is more evident in the mutant cells than in the wild-type cells.

**Figure 5:**
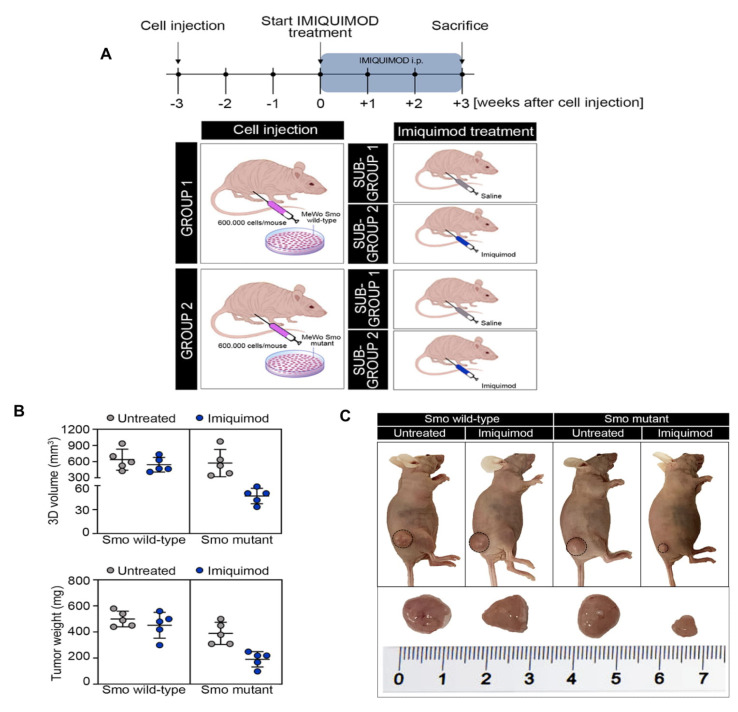
(**A**) Schematic representation of experimental strategy. Mice were injected subcutaneously in the right flank with MeWo cells expressing the SMO wild-type or mutant gene (600,000 cells/mouse), as described in Materials and Methods. After 3 weeks (average tumor size 300 mm^3^) mice were treated with IMQ (25 mg/kg, every other day, by i.p.) for 3 weeks; (**B**) 3D volume (mm^3^) and tumor weight (mg) of SMO wild-type and mutant-gene-amplified MeWo tumor xenografts, treated with vehicle or IMQ i.p. Data are means ± SD of five mice in each group; (**C**) Representative images of four group of mice enclosed into experimental plan: Group 1: SMO wild-type gene-amplified MeWo cell lines, devised into sub-group 1: vehicle control and sub-group 2: IMQ administrated by i.p.; Group 2: SMO mutant-gene-amplified MeWo cell lines, devised into sub-group 1: vehicle control and sub-group 2: IMQ administrated by i.p.

Moreover, we then evaluated whether the in vitro observations had relevance on the clinical response seen in the observed patient. Two groups of 10 nude mice were subcutaneously injected with the SMO wild-type and mutant-gene-amplified MeWo cell lines. Three weeks following injection, the mice were randomized into two groups and treated with a vehicle or IMQ (25 mg/kg) for 3 weeks every other day (Figure 5A). As shown in Figure 5B,C, IMQ treatment had little or no effect on tumor growth in SMO wild-type gene amplified tumor xenografts. In fact, the growth rate of tumors treated with IMQ was similar to those treated with the vehicle, reaching the maximum allowed tumor size, with a mean tumor volume of 620 and 590 mm^3^, respectively (Figure 5B,C). On the contrary, opposite results were obtained in the groups of SMO mutant-gene-amplified tumor xenografts, in which IMQ treatment led to a sharp anti-tumor efficacy, suppressing tumor growth at the end of the 3 weeks of treatment (Figure 5B,C). In particular, IMQ treatment caused a regression in the tumor size in tumor xenografts derived from SMO mutant-gene-amplified MeWo cells, with a mean tumor volume of 600 and 50 mm^3^, respectively (Figure 5B,C).

**Figure 6:**
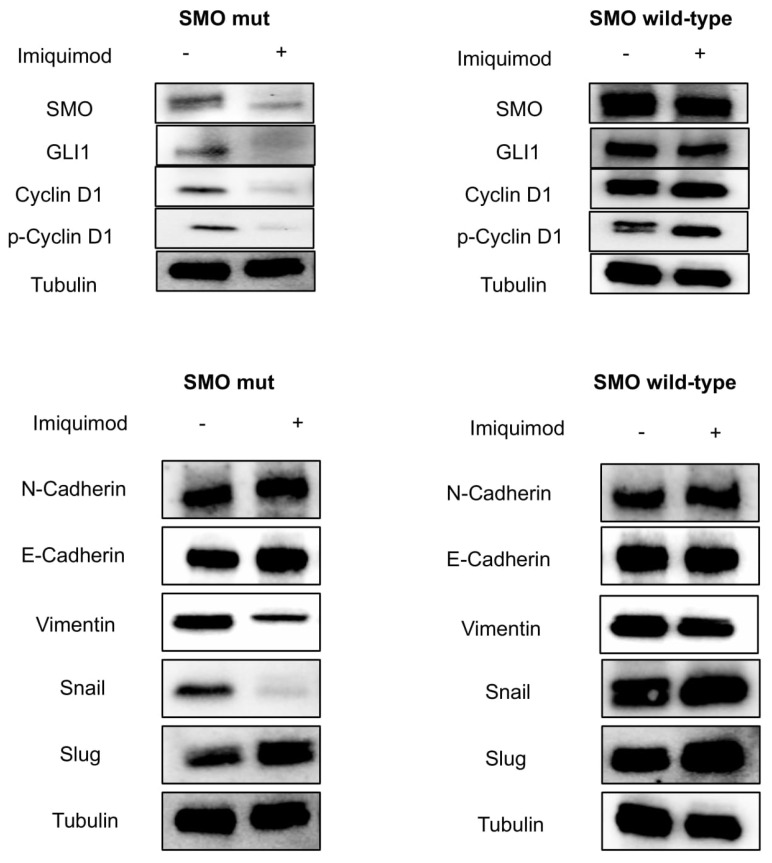
Tumor samples were collected, and total cell protein extracts were subjected to immunoblotting with the indicated antibodies (bar graphs in Supplementary Figure S4), as described in Materials and Methods. Anti-tubulin antibody was used for normalization of protein extract content.

To better demonstrate the role of IMQ in suppressing oncogenic the SMO signaling pathway independently of its immune modulating function, tumors were collected at the end of the treatment from mice engrafted with both SMO wild-type and mutant-gene-amplified MeWo cell lines. Finally, we assessed the direct effect of IMQ on the SMO signaling pathway by Western Blot analysis (Figure 6). These results underlined the SMO-related antitumor activity of IMQ in mutant models (Figure 6).

## 3. Materials and Methods

### 3.1. DNA Extraction

An appropriate formalin-fixed paraffin-embedded (FFPE) tissue block was selected for the patient. Three unstained FFPE tissue sections were cut at 10 μm each for DNA extraction. DNA was obtained using the QIAamp^®^ DNA FFPE Tissue (Qiagen, Hilden, Germany), according to the manufacturer’s instructions. Extracted DNA was eluted in 30 µL of elution buffer and then DNA was quantified by a Qubit^®^ 2.0 Fluorometer (Life Technologies) using the Qubit^®^ dsDNA HS Assay kit, according to the manufacturer’s recommendations. The extracted DNA was stored at −20 °C.

### 3.2. Next Generation Sequencing

Around 10 ng of DNA was used to prepare the sequencing libraries. The libraries were prepared with the IonAmpliSeq™ Library kit 2.0 (Thermo Fisher Scientific, Waltham, MA, USA) and with a primer pool (AmpliSeq Cancer Hotspot Panel v2 that analyses 2800 mutational hotspots and targeted regions in 50 genes (ABL1, EGFR, GNAS, KRAS, PTPN11, AKT1, ERBB2, GNAQ, MET, RB1, ALK, ERBB4, HNF1A, MLH1, RET, APC, EZH2, HRAS, MPL, SMAD4, ATM, FBXW7, IDH1, NOTCH1, SMARCB1, BRAF, FGFR1, JAK2, NPM1, SMO, CDH1, FGFR2, JAK3, NRAS, SRC, CDKN2A, FGFR3, IDH2, PDGFRA, STK11, CSF1R, FLT3, KDR, PIK3CA, TP53, CTNNB1, GNA11, KIT, PTEN, VHL). Amplified products were purified with Agencourt AMPure XP beads (Beckman Coulter Genomics, High Wycombe, UK). Concentrations of amplified and barcoded libraries were measured using the Qubit ^®^ 2.0 Fluorometer and the Qubit^®^ dsDNA HS Assay kit. DNA libraries were stored at −20 °C. The libraries were clonally amplified on Ion Sphere TM particles after dilution of the libraries to 100 pM. Template preparation was performed with the IonOneTouch™ 2 System (Thermo Fisher Scientific), an automated system for emulsion PCR, followed by recovery of Ion Sphere™ Particles, and enrichment of template-positive particles. The Ion Sphere™ particles coated with the template were applied to the semiconductor chip. A short centrifugation step was conducted to allow the spherical particles to be deposited into the chip wells. Finally, sequencing was carried out using Ion 314™ chips on the Ion Personal Genome Machine System (PGM™, Thermo Fisher Scientific) using the Ion PGM™ Hi-Q view Sequencing kit v2.

### 3.3. Methods of Sequence Analysis

The Torrent Suite Software v.4.0.2 (Life Technologies) was used to assess run performance and data analysis. The Integrative Genomics Viewer (IGV v 2.2, Broad Institute) was used for visual inspection of the aligned reads. Sequencing data were analyzed using Ion Reporter software (https://ionreporter.lifetechnologies.com/, 10 September 2020) and further filtered through quality checking. We selected all SNVs in the studied genes resulting in a non-synonymous amino acid change, or a premature stop codon, and all short indels resulting in either a frameshift or insertion/deletion of amino acids. All SNVs were analyzed for previously reported hotspot mutations (somatic mutations reported in COSMIC database) and novel variations, i.e., new mutations detected by NGS but not reported in either COSMIC or db SNP databases.

### 3.4. Cell Cultures and Transfections

A375 cells were purchased from the American Type Culture Collection (ATCC). Cells were cultured in Dulbecco’s modified Eagle’s Medium (DMEM), or RPMI, both supplemented with 10% FBS and 1% penicillin/streptomycin. MeWo cells were purchased from the ATCC. Cells were grown in a DMEM medium, supplemented with 10% fetal bovine serum, 1% L-Glutamine and 1% Penicillin/Streptomycin, and maintained in a humidified controlled atmosphere at 37 °C. The MeWo cell lines were transfected with 5.0 μg of wild-type or p.Gln216Arg mutant SMO Protein Vector (Human) (pPM-N-D-C-His, abm) using Lipofectamine-2000 (Invitrogen™, Carlsbad, CA, USA), according to the manufacturer’s instructions, to generate their SMO wild-type and mutant gene amplified MeWo cell line. After 48 h, Geneticin^®^ (GIBCO by Life Technologies), at a final concentration of 0.8 mg/mL, was used to select Geneticin-resistant cells.

### 3.5. Site-Directed Mutagenesis in SMO Protein Vector (Human) (pPM-N-D-C-His)

SMO Protein Vector (pPM-N-D-C-His, abm Cat.No. PV434509, Accession Number BC009989) was purchased by Applied Biological Materials Inc. (abm, Richmond, BC, Canada). The p.Gln216Arg mutation was introduced in the SMO Protein Vector, by PCR site-directed mutagenesis, by using primers which contain one base that differs from the wild-type sequence (SMO wild-type CAG Glu216, SMO mutant CGG Arg216). Oligos were designed and ordered from Eurofins Genomics. The sequences of oligonucleotides used for PCR site-directed mutagenesis are shown in Supplementary Table S1. PCR site-directed mutagenesis was performed by using a version of the high-fidelity DNA polymerase, i.e., Q5 DNA polymerase (New England Biolabs, Ipswich, MA, USA) in order to ensure accurate amplification. The PCR product carrying the mutation of interest was cleaved with the XmaI restriction enzyme (New England BioLabs, MA, USA) and inserted in place of the wild-type sequence into the SMO Protein Vector (digested with XmaI restriction enzyme). A schematic diagram for site-directed mutagenesis is shown in Figure 2B. SMO Protein Vector mutagenesis was then confirmed by DNA sequencing.

### 3.6. Real-Time PCR

Messenger RNAs were extracted with the TRIzol reagent (Invitrogen™). Complementary DNAs were prepared with the 5X All-In-One RT MasterMix (Applied Biological Materials Inc., abm), as indicated by the manufacturer instructions. The cDNAs were amplified by PCR in a CFX Connect Real-Time PCR Detection System (BioRad, Hercules, CA, USA) with the fluorescent double-stranded DNA-binding dye SYBR Green (BioRad). Specific primers for each gene were designed to work under the same cycling conditions (95 °C for 10 min followed by 40 cycles at 95 °C for 15 s and 60 °C for 1 min), thereby generating products of comparable sizes (about 200 bp for each amplification). Primer combinations were positioned, whenever possible, to span an exon–exon junction and the RNA digested with DNAse to avoid genomic DNA interference. For each reaction, standard curves for reference genes were constructed based on six four-fold serial dilutions of cDNA. All samples were run in triplicate. The template concentration was calculated from the cycle number when the amount of PCR product passed a threshold established in the exponential phase of the PCR. The relative amounts of gene expressions were calculated with Cyclophilin-A expression as an internal standard (calibrator). The results, expressed as N-fold differences in target gene expression, were determined as follows: N * target = 2^(ΔCt sample-ΔCt calibrator)^.

### 3.7. Cell Viability Assay

MeWo cells lines were seeded into 24-well plates at the density of 1 × 104 cells/well and were exposed to different concentrations of imiquimod (range, 0.05–25 µM) for 72 h. Cell viability was measured with the 3-(4,5-dimethylthiazol-2-yl)-2,5-diphenyl-tetrazolium bromide (MTT) solution (Sigma-Aldrich). The MTT solution was removed and formazan crystals were extracted with isopropanol supplemented with 1% of HCl (200 µL/well). The 24-well plates were stirred up for 10 min and, subsequently, 100 µL of solution was transferred into a 96-well. Absorbance of the formazan’s solution in isopropanol-HCl was measured spectrophotometrically at a wavelength of 550 nm. The IC50 value was determined by interpolation from the dose–response curves. The results represent the median of three separate experiments, each performed in duplicate.

### 3.8. Western Blot Assay

MeWo human melanoma cell lines, parental and transfected (mutants and wild-type), were treated with imiquimod at dose of 1 μM for 24 h. Tumor samples were harvested from euthanized mice, cut into 20 to 25 mm^3^ pieces, and frozen at −80 °C. Subsequently, frozen samples were homogenized in a RIPA buffer, supplied by protease and phosphatase inhibitors, using an Ultra-turrax homogenizer. Tissue lysates were clarified by centrifugation at 14,000 rpm for 10 min a 4 °C. The protein concentration was determined using a Bradford assay (Bio-Rad), and equal amounts of proteins were separated by SDS-PAGE gel and transferred to a nitrocellulose membrane (Bio-Rad). Equal amounts of total proteins were incubated with the following primary polyclonal antibodies: SMO, SUFU, GLI1, Bcl-2, Bcl-xl, BAX, PARP, Caspase-8, Caspase-3, Slug, Snail, Vimentin and N-Cadherin. SMO was from Santa Cruz (SC-166685). All other antibodies are purchased from Cell Signaling. The monoclonal anti-α-tubulin antibody (Sigma-Aldrich) was used as loading control antibody. Secondary antibodies, goat anti-rabbit IgG and rabbit anti-mouse IgG (GOAT X RABBIT-HRP 1706515, GOAT X Mouse-HRP 1706516), were from Bio-rad (Hercules, CA, USA). The primary antibodies were diluted in a blocking solution (1X TBS and 0.1% Tween 20) composed of 5% *w/v* BSA or 5% *w/v* nonfat dry milk, according to the specifications on the product analysis certificate (dilution 1:1000). The secondary antibodies were diluted in a blocking solution of 5% *w/v* nonfat dry milk (dilution 1:3000). After incubation with the secondary anti-mouse antibody, membranes were developed using an enhanced chemi-luminescence (ECL) detection system (BioRad).

### 3.9. Apoptosis Assay

Mewo parental and transfected cell lines were seeded in 6-well plates and treated with imiquimod at a dose of 1 µM for 24 h. Cells were harvested with trypsin, washed with PBS and suspended in a binding buffer (Invitrogen, USA). Subsequently, 5 µL of Annexin V-FITC (Invitrogen) and 5 µL of propidium iodide (Invitrogen) were added into the cell’s suspension and incubated at room temperature in the dark for 30 min. The analysis was performed by using the BD Accuri C6 Flow Cytometer (BD Biosciences, San Jose, CA, USA). Histogram analysis was performed by using the GraphPad Prism software.

### 3.10. Tumor Xenografts in Nude Mice

Five-week-old female athymic (nu/nu) mice were purchased from ENVIGO Laboratories. After one week of adaptation, 20 animals were randomized into two group and half of the mice (10 mice/group) were subcutaneously injected in the right flank with a suspension of 6.0 × 10^5^ SMO wild-type or mutant-gene-amplified MeWo cells, in 50 μL Dulbecco’s Modified Eagle Medium (Life Technologies, Carlsbad, CA, USA) + 50 μL Matrigel Growth Factor Reduced (GFR) Basement Membrane Matrix (Corning), for a total volume of 100 μL. GROUP 1: SMO wild-type gene-amplified MeWo cells; GROUP 2: SMO mutant gene amplified MeWo cells. Tumor size was measured continuously to follow the development of tumors and monitor the health of the mice. After 3 weeks post-injection, each group of injected mice was randomized into two sub-groups (five mice/sub-group) and treated with vehicle or IMQ (25 mg/kg) for 3 weeks, every other day. SUB-GROUP 1: vehicle administrated intraperitoneally (i.p.); SUB-GROUP 2: IMQ administrated intraperitoneally (i.p.). Tumor volume was measured using the formula π/6 × larger diameter x (smaller diameter)^2^. Tumors were collected when reaching a size of 620 and 590 mm^3^ in SMO wild-type gene amplified MeWo cells and 600 and 50 mm^3^ in SMO mutant gene amplified MeWo cells, respectively.

## 4. Discussion

The HH signaling pathway plays a crucial role during embryonic development, tissue homeostasis, and regeneration [16,19]. Aberrant activation of the HH pathway has been reported to drive tumor progression in several cancers, including those of the skin, brain, lung, pancreas, stomach, and hematopoietic malignancies [16,19]. SMO is one of the major components of the HH pathway [14]. HH ligand binding to Patched (PTCH) inhibits the repressive function of PTCH, allowing SMO to transduce the signal and triggering of an intracellular cascade that ultimately leads to the activation of the GLI transcription factors [14,15,16]. Recently, several groups have described the important role of the HH pathway in melanoma progression [20,21]. In particular, O’ Really KE et al., in their work, have demonstrated that HH pathway inhibition might be a promising targeted therapy in selected metastatic melanoma patients. [22]. IMQ is an immune modulator agent approved for the treatment of superficial basal cell carcinoma (BCC). Its anti-tumor effect has not been well described. Gruber et al. investigated the possibility of IMQ inhibiting the HH/GLI pathway and discuss the possible benefits for the treatment of HH-associated cancer [23]. Here we report, for the first time to our knowledge, a clinical case where a melanoma patient harboring the SMO p.Gln216Arg mutation has been treated with IMQ. The patient carrying the p.Gln216Arg mutation in SMO showed a complete response. The brilliant response obtained was still present after 32 months, highlighting a novel mechanism of action independent of the immune modulator effect of IMQ. This clinical case represents an advance in precision medicine fields, opening a new scenario for the treatment of melanoma patients, identifying new targets and new biomarkers to be further impacted by therapeutics.

## Data Availability

Not relevant.

## Supplementary Materials

The following are available online at https://www.mdpi.com/2075-4426/11/3/206/s1, Figures S1–S4; Table S1: List of oligonucleotides used for PCR site-directed mutagenesis.

## References

[1] Bray F., Ferlay J., Soerjomataram I., Siegel R.L., Torre L.A., Jemal A. (2018). Global cancer statistics 2018: GLOBOCAN estimates of incidence and mortality worldwide for 36 cancers in 185 countries. CA Cancer J. Clin..

[2] Leonardi G.C., Falzone L., Salemi R., Zanghì A., Spandidos D.A., Mccubrey J.A., Candido S., Libra M. (2018). Cutaneous melanoma: From pathogenesis to therapy (Review). Int. J. Oncol..

[3] Weide B., Faller C., Büttner P., Pflugfelder A., Leiter U., Eigentler T.K., Bauer J., Forschner A., Meier F., Garbe C. (2013). Prognostic factors of melanoma patients with satellite or in-transit metastasis at the time of stage III diagnosis. PLoS ONE.

[4] Marks R., Gebauer K., Shumack S., Amies M., Bryden J., Fox T.L., Owens M.L. (2001). Imiquimod 5% cream in the treatment of superficial basal cell carcinoma: Results of a multicenter 6-week dose-response trial. J. Am. Acad. Dermatol..

[5] Geisse J., Caro I., Lindholm J., Golitz L., Stampone P., Owens M. (2004). Imiquimod 5% cream for the treatment of superficial basal cell carcinoma: Results from two phase III, randomized, vehicle-controlled studies. J. Am. Acad. Dermatol..

[6] Rivas-Tolosa N., Ortiz-Brugués A., Toledo-Pastrana T., Baradad M., Traves V., Soriano V., Sanmartín V., Requena C., Marti R., Nagore E. (2015). Local cryosurgery and imiquimod: A successful combination for the treatment of locoregional cutaneous metastasis of melanoma: A case series. J. Dermatol..

[7] Shi V.Y., Tran K., Patel F., Leventhal J., Konia T., Fung M.A., Wilken R., Garcia M.S., Fitzmaurice S.D., Joo J. (2015). 100% Complete response rate in patients with cutaneous metastatic melanoma treated with intralesional interleukin (IL)-2, imiquimod, and topical retinoid combination therapy: Results of a case series. J. Am. Acad. Dermatol..

[8] Florin V., Desmedt E., Vercambre-Darras S., Mortier L. (2012). Topical treatment of cutaneous metastases of malignant melanoma using combined imiquimod and 5-fluorouracil. Investig. New Drugs.

[9] Schön M. (2007). Imiquimod: Mode of action. Br. J. Dermatol..

[10] Wolff F., Loipetzberger A., Gruber W., Esterbauer H., Aberger F., Frischauf A.M. (2013). Imiquimod directly inhibits Hedgehog signalling by stimulating adenosine receptor/protein kinase A-mediated GLI phosphorylation. Oncogene.

[11] Sanderson P., Innamaa A., Palmer J., Tidy J. (2013). Imiquimod therapy for extramammary Paget’s disease of the vulva: A viable non-surgical alternative. J. Obstet. Gynaecol..

[12] Peris K., Micantonio T., Fargnoli M.C., Lozzi G.P., Chimenti S. (2006). Imiquimod 5% Cream in the Treatment of Bowen’s Disease and Invasive Squamous Cell Carcinoma. J. Am. Acad. Dermatol..

[13] Yokoyama M., Egawa G., Makino T., Egawa K. (2019). Erythroplasia of Queyrat treated with imiquimod 5% cream: The necessity of regimen guidelines. Clin. Case Rep..

[14] Yang L., Xie G., Fan Q., Xie J. (2009). Activation of the hedgehog-signaling pathway in human cancer and the clinical implications. Oncogene.

[15] Onishi H., Kai M., Odate S., Iwasaki H., Morifuji Y., Ogino T., Morisaki T., Nakashima Y., Katano M. (2011). Hypoxia activates the hedgehog signaling pathway in a ligand-independent manner by upregulation of Smo transcription in pancreatic cancer. Cancer Sci..

[16] Athar M., Tang X., Lee J.L., Kopelovich L., Kim A.L. (2006). Hedgehog signalling in skin development and cancer. Exp. Dermatol..

[17] Yoo Y.A., Kang M.H., Lee H.J., Kim B.-H., Park J.K., Kim H.K., Kim J.S., Oh S.C. (2011). Sonic hedgehog pathway promotes metastasis and Lymphangiogenesis via activation of Akt, EMT, and MMP-9 pathway in gastric cancer. Cancer Res..

[18] Ahmad A., Maitah M.Y., Ginnebaugh K.R., Li Y., Bao B., Gadgeel S.M., Sarkar F.H. (2013). Inhibition of Hedgehog signaling sensitizes NSCLC cells to standard therapies through modulation of EMT-regulating miRNAs. J. Hematol. Oncol..

[19] Rubin L.L., de Sauvage F.J. (2006). Targeting the hedgehog pathway in cancer. Nat. Rev. Drug Discov..

[20] Stecca B., Mas C., Clement V., Zbinden M., Correa R., Piguet V., Beermann F., i Altaba A.R. (2007). Melanomas require HEDGEHOG-GLI signaling regulated by interactions between GLI1 and the RAS-MEK/AKT pathways. Proc. Natl. Acad. Sci. USA.

[21] Alexaki V.I., Javelaud D., Van Kempen L.C., Mohammad K.S., Dennler S., Luciani F., Hoek K.S., Juàrez P., Goydos J.S., Fournier P.J. (2010). GLI2-mediated melanoma invasion and metastasis. J. Natl. Cancer Inst..

[22] O’Reilly K.E., De Miera E.V.-S., Segura M.F., Friedman E., Poliseno L., Han S.W., Zhong J., Zavadil J., Pavlick A., Hernando E. (2013). Hedgehog pathway blockade inhibits melanoma cell growth in vitro and in vivo. Pharmaceuticals.

[23] Gruber W., Frischauf A.M., Aberger F. (2014). An old friend with new skills: Imiquimod as novel inhibitor of Hedgehog signaling in basal cellcarcinoma. Oncoscience.

