# Supplementary material for: Treatment of Cutaneous Melanoma Harboring SMO p.Gln216Arg Mutation with Imiquimod: An Old Drug with New Results
Source: J Pers Med. 2021 Mar 14;11(3):206. doi: 10.3390/jpm11030206 (PMC8000647; doi:10.3390/jpm11030206)
Supplement: Supplementary file 1 [file jpm-11-00206-s001.zip › Supplementary Figures S1-S4.pdf]

Figure 1

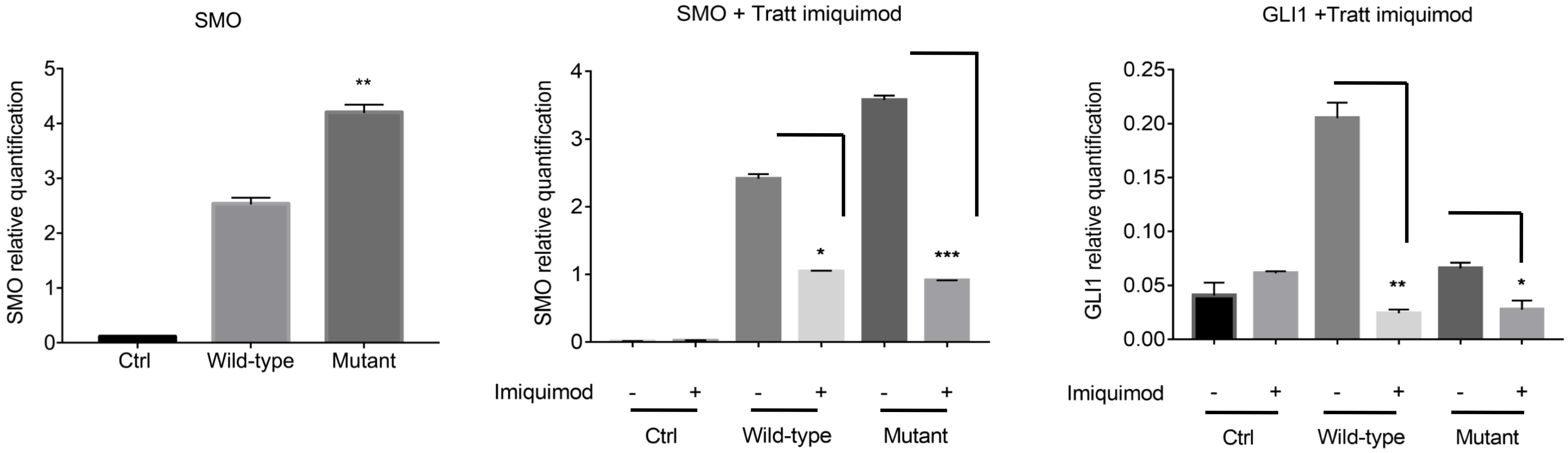

One-way ANOVA

** Wild-type vs mutant p value 0.0055

T-test

* Ctrl vs treated p value 0.02

*** Ctrl vs treated p value 0.0003

T-test

** Ctrl vs treated p value 0.003

* Ctrl vs treated p value 0.0323

Figure 2

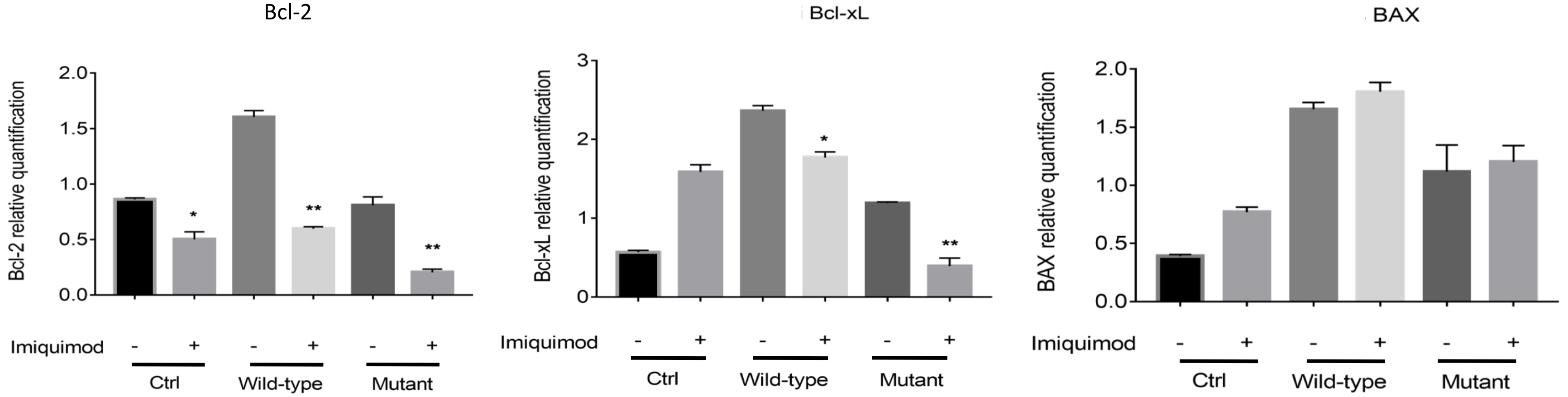

T-test

* Ctrl vs treated p value 0.018

** Ctrl vs treated p value 0.0017

** Ctrl vs treated p value 0.0090

T-test

* Ctrl vs treated p value 0.013

** Ctrl vs treated p value 0.0081

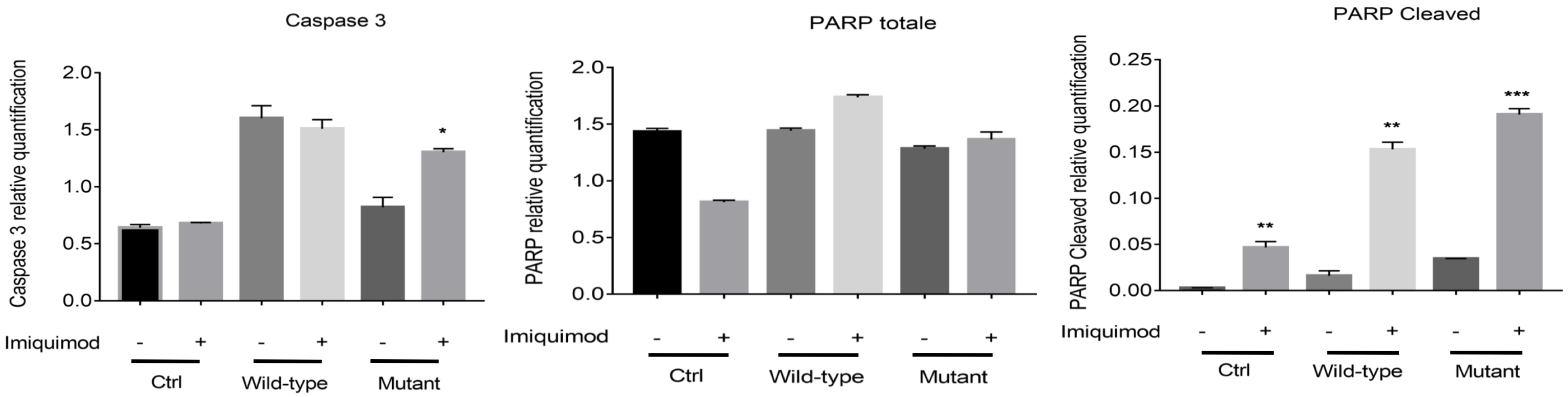

T-test

* Ctrl vs treated p value 0.0179

T-test

** Ctrl vs treated p value 0.0098

** Ctrl vs treated p value 0,0023

*** Ctrl vs treated p value 0,0008

Figure 3

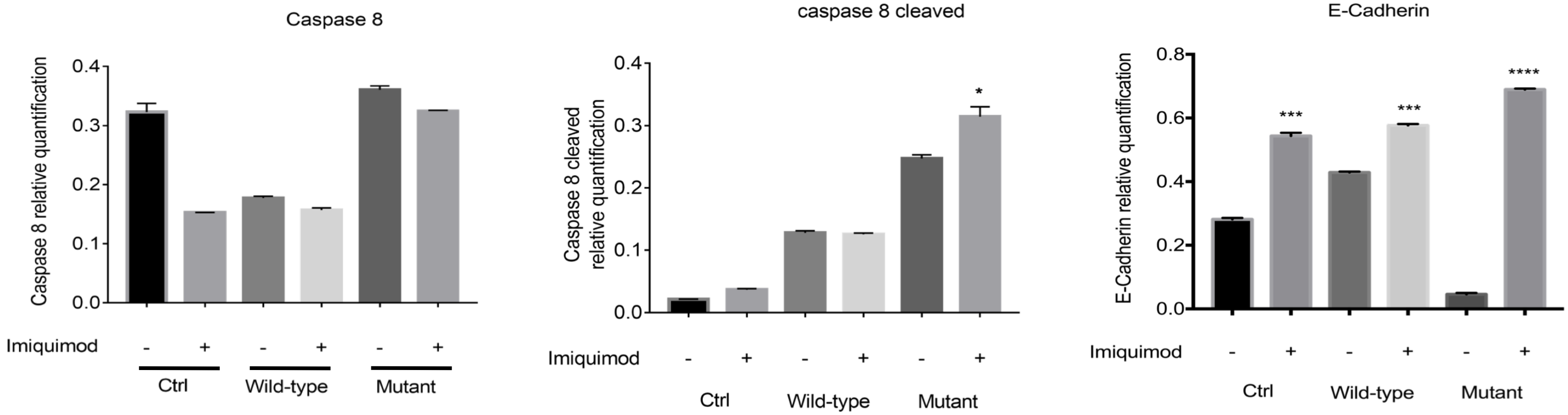

T-test

* Ctrl vs treated p value 0.0307

T-test

*** Ctrl vs treated p value 0.0009

*** Ctrl vs treated p value 0.0007

**** Ctrl vs treated p value <0.0001

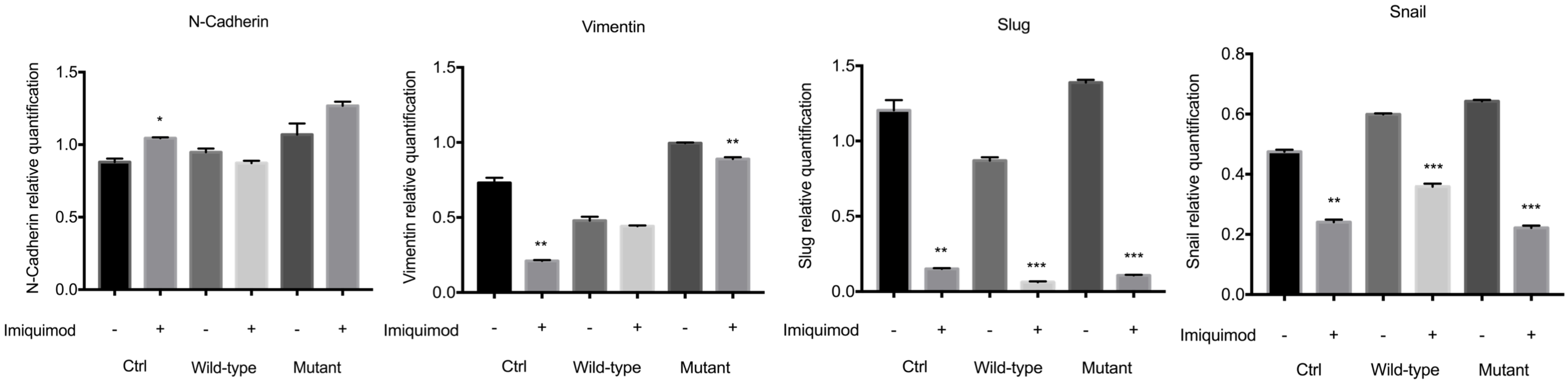

T-test

* Ctrl vs treated p value 0.011

T-test

** Ctrl vs treated p value 0.0022

** Ctrl vs treated p value 0.0084

T-test

** Ctrl vs treated p value 0.0021

*** Ctrl vs treated p value 0.0004

*** Ctrl vs treated p value 0.0001

T-test

** Ctrl vs treated p value 0.0011

*** Ctrl vs treated p value 0.0010

*** Ctrl vs treated p value 0.0002

Figure 4

Tumor xenografts in nude mice

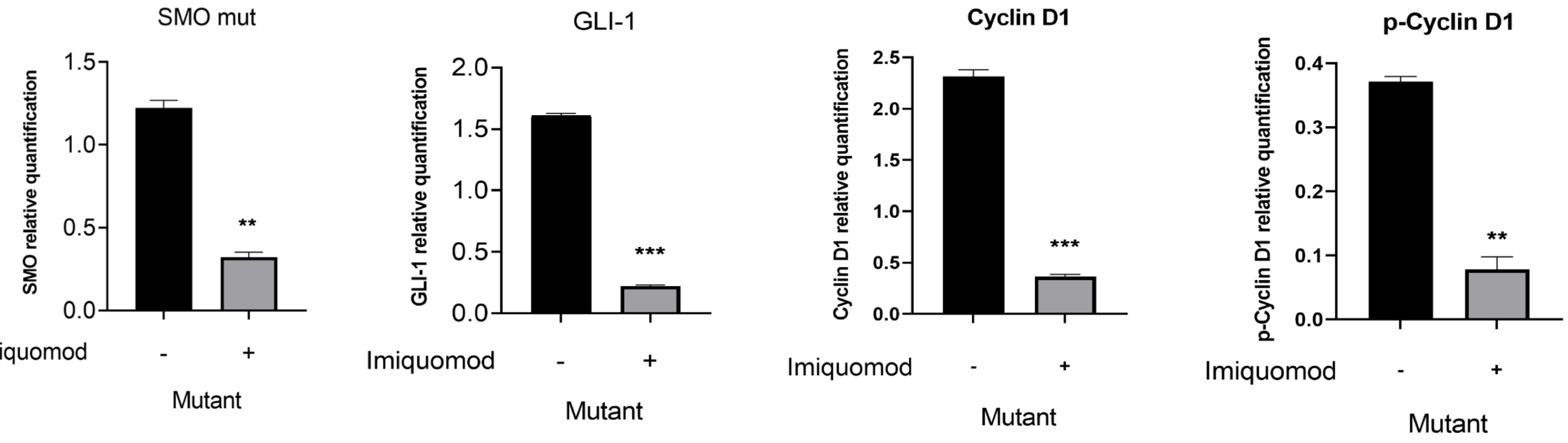

T-test

** Ctrl vs treated p value 0.0019

T-test

*** Ctrl vs treated p value 0.0001

T-test

*** Ctrl vs treated p value 0.0006

T-test

** Ctrl vs treated p value 0.0026

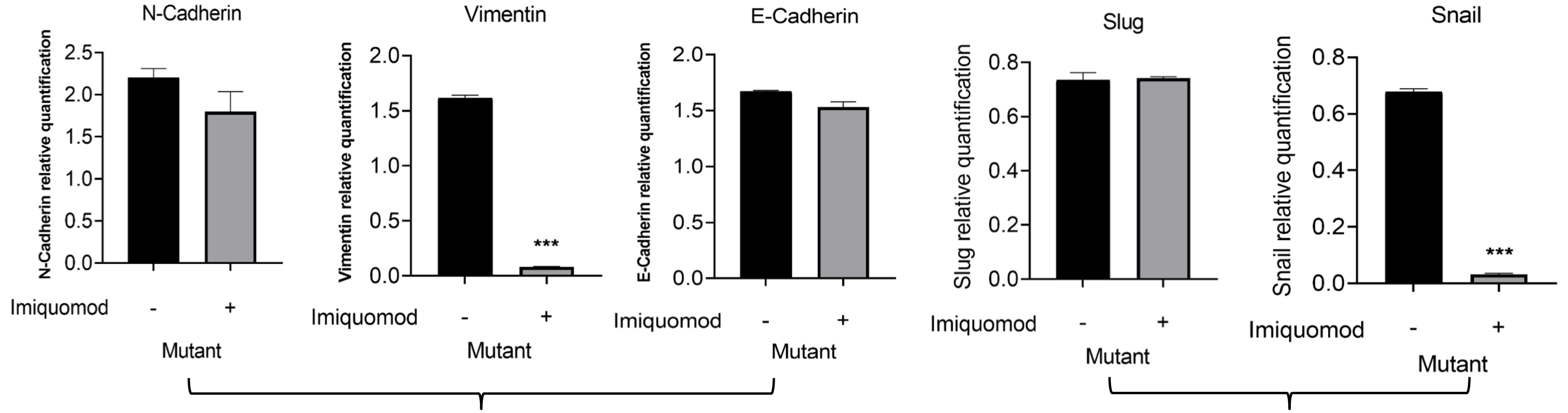

T-test

*** Ctrl vs treated p value 0.0001

T-test

*** Ctrl vs treated p value 0.0001
