# Supplementary material for: Treatment of Cutaneous Melanoma Harboring SMO p.Gln216Arg Mutation with Imiquimod: An Old Drug with New Results
Source: J Pers Med. 2021 Mar 14;11(3):206. doi: 10.3390/jpm11030206 (PMC8000647; doi:10.3390/jpm11030206)
Supplement: Supplementary file 1 [file jpm-11-00206-s001.zip › Supplementray Table S1.docx]

**Supplementary Table S1:** List of oligonucleotides**.**

| **Oligonucleotides used for Real-Time PCR** | | |
| --- | --- | --- |
| **Oligo** | **Sense** | **Sequence** |
| **Cyclophilin A** | Forword | AGTCCATCTATGGGGAGAAATTTG |
|  | Reverse | GCCTCCACAATATTCATGCCTTC |
| **E-Cadherin** | Forword | GGCGCCACCTCGAGAGA |
|  | Reverse | TGTCGACCGGTGCAATCTT |
| **GLI1** | Forword | TCCACAGGCATACAGGATCC |
|  | Reverse | GATGTGCTCGCTGTTGATGT |
| **GLI2** | Forword | CCTACCGATTGACATGCGAC |
|  | Reverse | ACAGAACGGAGGTAGTGCTC |
| **N-Cadherin** | Forword | ACAGTGGCCACCTACAAAGG |
|  | Reverse | CCGAGATGGGGTTGATAATG |
| **SMO** | Forword | TCATCGTGGGAGGCTACTTC |
|  | Reverse | GGCAGCTGAAGGTAATGAGC |
| **Snail** | Forword | ATGCACATCCGAAGCCACA |
|  | Reverse | GACTCTTGGTGCTTGTGGAG |
| **Vimentin** | Forword | GAACCTGCAGGAGGCAGAAG |
|  | Reverse | CATCTTAACATTGAGCAGGTC |
| **Oligonucleotides used for SMO point mutation** | | |
| **Smo-clon** | Forword | TGTACAAAAAAGTTGGCACC |
|  | Reverse | CAGCCTTCTCACTCAGCAGC |
| **Smo-mut** | Forword | CTGCGGCATCCGGTGCCAGAAC |
|  | Reverse | GTTCTGGCACCGGATGCCGCAG |
